# Effect of DNA Repair Protein Rad18 on Viral Infection

**DOI:** 10.1371/journal.ppat.0020040

**Published:** 2006-05-19

**Authors:** Aliza G Lloyd, Satoshi Tateishi, Paul D Bieniasz, Mark A Muesing, Masaru Yamaizumi, Lubbertus C. F Mulder

**Affiliations:** 1 The Aaron Diamond AIDS Research Center, The Rockefeller University, New York, New York, United States of America; 2 Institute of Molecular Embryology and Genetics, Kumamoto University, Kumamoto, Japan; King's College London, United Kingdom

## Abstract

Host factors belonging to the DNA repair machineries are assumed to aid retroviruses in the obligatory step of integration. Here we describe the effect of DNA repair molecule Rad18, a component of the post-replication repair pathway, on viral infection. Contrary to our expectations, cells lacking Rad18 were consistently more permissive to viral transduction as compared to Rad18^+/+^ controls. Remarkably, such susceptibility was integration independent, since retroviruses devoid of integration activity also showed enhancement of the initial steps of infection. Moreover, the elevated sensitivity of the Rad18^−/−^ cells was also observed with adenovirus. These data indicate that Rad18 suppresses viral infection in a non-specific fashion, probably by targeting incoming DNA. Furthermore, considering data published recently, it appears that the interactions between DNA repair components with incoming viruses, often result in inhibition of the infection rather than cooperation toward its establishment.

## Synopsis

Various cellular factors are thought to interact with invading retroviruses, either assisting the viral proteins or functioning as protecting factors against them. Productive viral infection, therefore, depends on the way the equilibrium between these two opposite group of molecules is leaning. The authors find that Rad18, a component of the post-replication DNA repair pathway, instead of aiding retroviral infection suppresses its establishment, interfering with the accumulation of the invading DNA. They also find that such suppressive activity is not restricted only to retroviruses but also concerns adenovirus infection. These results, in conjunction with those recently published, lead the authors to hypothesize a role for DNA repair mediated genome stability maintenance in viral infection, or in other words, that viral invasion is also a matter of genome stability.

## Introduction

The life cycle of retroviruses distinguishes itself from that of other viruses by the fact that it undergoes a process of reverse transcription and insertion of its genome into that of the host. This latest step is carried out by viral integrase (IN) in concert with host proteins most likely to be part of the various DNA repair machineries. A number of physical and/or functional interactions have been described between the viral constituents involved with the integration process and cellular elements that may act as cofactors for catalysis or gap repair, reviewed by Turlure et al. [1]. Mechanisms that have been proposed to be involved in DNA repair and DNA metabolism during retroviral integration include: base excision repair [2,3], homologous recombination (HR) [4] and non-homologous end joining [5–8]. Furthermore Lau et al. [9] have recently reported that the HR molecule Rad52 is a suppressor of HIV-1 infection, but ruled out a direct role of HR, as other proteins of this pathway did not affect retroviral transduction.

We have previously described that Rad18, a molecule belonging to a distinct DNA repair pathway known as post-replication DNA repair, associates with HIV-1 integrase when both proteins are overexpressed in human embryonic kidney (HEK) 293T cells [10]. Under these conditions, such association results in the stabilization of integrase, a substrate of the proteasome N-end rule pathway [11,12], as well as the co-localization of IN and Rad18 in nuclear structures.

Studies on the mechanism of post-replication DNA repair, both in yeast and mammalian cells, have shown that Rad18 is directly responsible for the specific mono-ubiquitylation of the polymerase adapter PCNA [13–16]. These reports show strong evidence of the preferential binding of polymerase η to mono-ubiquitylated PCNA, and that such binding takes place in replication foci in a Rad18-dependent fashion [15,16]. The absence of Rad18 in yeast has also been identified as the cause of gross chromosomal rearrangements [17], whereas in chicken and in mouse cells, Rad18 deficiency results in genome instability, specifically in an increase of sister chromatid exchange [18,19]

Here we present the results of a genetic study on the relevance of Rad18 in the early phases of viral infection. We show that Rad18 suppresses viral infection. Moreover, we determined that Rad18 inhibitory activity is not restricted to retroviruses, but extends to adenovirus as well. Furthermore, we provide evidence that suggests that Rad18 acts on the viral DNA, preventing it from reaching the replicative stage.

## Results

### Rad18 Influences the Susceptibility to Retroviral Infection

**Figure 1:**
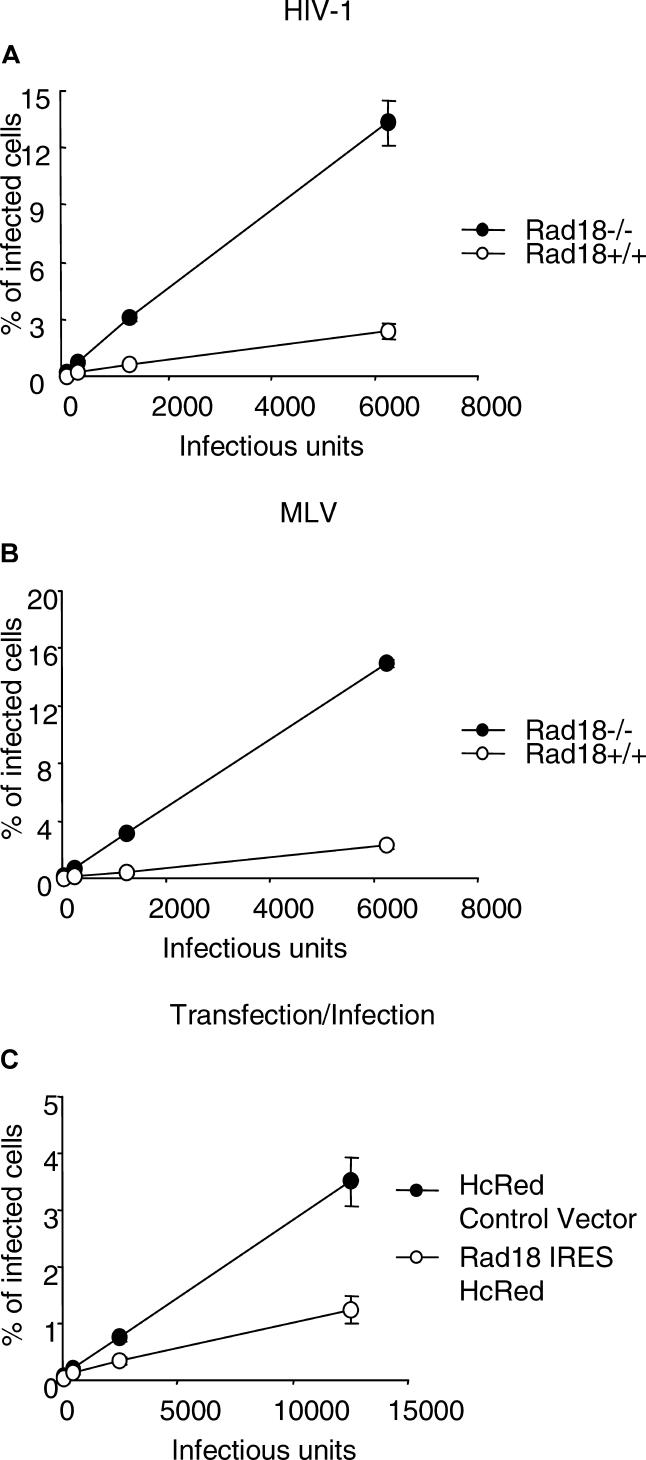
Rad18 Influences the Susceptibility to Retroviral Infection (A) Rad18^−/−^ MEFs, demonstrate a greater sensitivity to an HIV-1–based EGFP reporter virus infection as compared to wild type. Cells were infected with serial dilutions of an HIV-1–based EGFP reporter virus and assessed after 40–44 h by flow cytometry. Virus titers (IU) were measured in NIH3T3 cyc.T cells. (B) Rad18^−/−^ MEFs demonstrate a greater sensitivity to infection with MLV vector–derived virus as compared to wild type. MLV-based EGFP reporter virus was used to infect cells that were then assessed by flow cytometry 40–44 h later. (C) Hela cells expressing human Rad18 are more resistant to HIV-1 infection as compared to cells transfected with control plasmid. Cells transiently transfected with either hRad18-IRES-HcRed vector or IRES-HcRed control vector were infected with serial dilutions of a HIV-1–based EGFP reporter virus and assessed after 40–44 h by flow cytometry.

Because we previously found that Rad18 interacts with HIV-1 IN [10], we set out to investigate the susceptibility of cells devoid of Rad18 to HIV-1 infection. RAD18^−/−^ primary murine embryonic fibroblasts (MEFs) [16] and their wild-type counterpart were inoculated with increasing amounts of a vesicular stomatitis virus protein G (VSV-G) pseudotyped HIV-1–based enhanced green fluorescent protein (EGFP) reporter virus [20]. Analysis by flow cytometry for EGFP expression showed, surprisingly, that the Rad18^−/−^ cells were approximately 5-fold more sensitive to infection than Rad18^+/+^ cells (*t* test *p*-value = 0.007) (Figure 1A).

To investigate whether the enhanced susceptibility to infection of the Rad18^−/−^ cells was specific to HIV-1, we infected Rad18^−/−^ and Rad18^+/+^ cells with murine leukemia virus (MLV). Cells were inoculated with increasing amounts of an ecotropic envelope pseudotyped MLV-based retroviral vector expressing EGFP as reporter. Figure 1B shows that cells lacking Rad18 displayed approximately a 6-fold higher rate of infection as compared to Rad18^+/+^ cells (*t* test *p*-value = 7.795E−06).

Because we failed to obtain stable Rad18^−/−^ MEF cell lines complemented with Rad18, most likely due to the toxicity of Rad18 long-term unregulated expression, we next tested whether transient expression of Rad18 in human cells would inhibit infection. Hela were cells transfected with a bicistronic vector encoding Rad18-IRES-HcRed or as a control IRES-HcRed only, and infected with HIV-1–based EGFP reporter virus. Figure 1C shows that Rad18 overexpressing cells are markedly more resistant to HIV-1 infection (*t* test *p*-value = 0.017) as compared to cells transfected with control plasmid.

Taken together, these results indicate that Rad18 has an inhibitory effect on retroviral infection.

### The Increase in Infection of Rad18^−/−^ Cells Is Independent of Integrase Activity and Is Not Dependent on Illegitimate Integration

**Figure 2:**
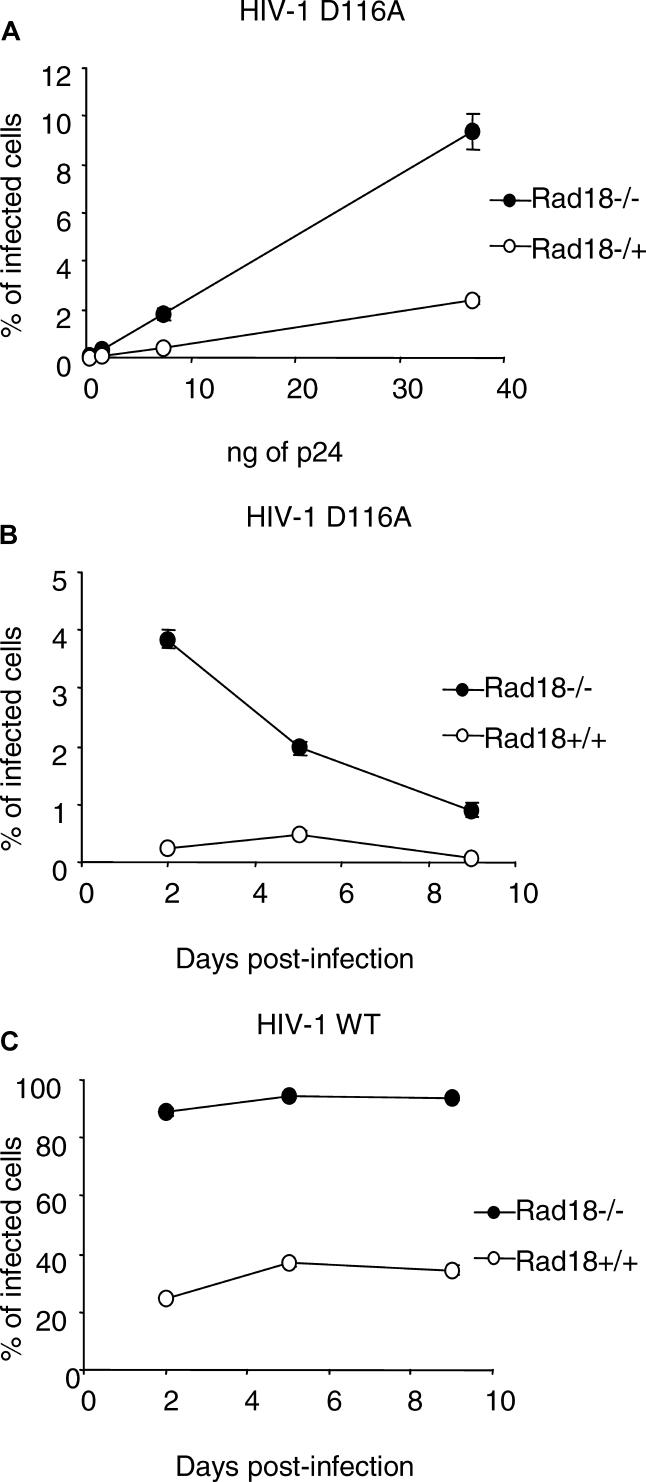
The Increase in Infection of Rad18^−/−^ Cells Is Independent of Integrase Activity and Is Not Dependent on Illegitimate Integration (A) Rad18^−/−^ MEFs show a greater susceptibility to infection with an integration-inert HIV-1 virus as compared to Rad18^+/+^ cells. Cells were infected with an integration-deficient HIV-1–based EGFP reporter virus using serial dilutions of p24 values equivalent to 125,000 IU of wild-type virus and then assessed after 40–44 h by flow cytometry. (B) MEFs Rad18^−/−^ and Rad18^+/+^ infected with an integration-deficient HIV-1–based EGFP reporter virus were assessed by flow cytometry at 2, 5, and 9 d after infection. (C) MEFs Rad18^−/−^ and Rad18^+/+^ infected with HIV-1–based EGFP reporter virus and assessed by flow cytometry at 2, 5, and 9 d after infection.

Rad18^−/−^ cells have a high frequency of sister chromatid exchange, and more efficiently incorporate selectable markers by stable transfection [19]. Given our previous findings, we asked if the increased susceptibility to infection in these cells was integrase dependent or if a higher integration frequency could occur in the Rad18^−/−^ cells even in the absence of a catalytically active viral integrase, facilitated by the absence of a functional post-replication DNA repair pathway. To test this assumption, cells were inoculated with HIV-1–based VSV-G pseudotyped reporter viruses with integrase harboring a mutation in the catalytic domain at the position D116 [21]. Figure 2 shows that the proportion of EGFP-positive cells was indeed 4-fold (*t* test *p-*value = 0.00042) higher in Rad18 knockout cells than in RAD18^+/+^, suggesting that the increase in infection of Rad18^−/−^ cells is independent of integrase catalytic activity.

In order to assess whether the EGFP expressed in cells infected with a mutant virus originated from integrase-independent integrated provirus or from non-integrated episomal viral DNA, Rad18^+/+^ and Rad18^−/−^ cells were inoculated with a HIV-1 retroviral vector–derived virus harboring a catalytically inactive integrase and split 1:10 at day 2, 5, and 9 post-infection. At the same time points, aliquots of the infected cells were analyzed by flow cytometry for EGFP expression. Figure 2B shows the frequency of EGFP-positive cells over time in RAD18^+/+^ and in Rad18^−/−^ cells. Rad18^+/+^ EGFP-positive cells are barely detectable, whereas Rad18^−/−^ cells show a higher frequency of positive cells, with a steady time-dependent decline. These results show that EGFP expression from IN-defective virus originates from non-integrated viral cDNA that may accumulate in greater quantities in Rad18^−/−^ than in Rad18^+/+^ and is then lost by dilution during cell division.

In parallel, we infected Rad18^+/+^ and Rad18^−/−^ cells with saturating amounts (about 250,000 infectious units [IU]) of HIV-1–derived retroviral vector–carrying wild-type integrase to test whether the absence of Rad18 could cause upon infection premature death as the result of the failure to repair the integration site. Cells were split and analyzed 2, 5, and 9 days post-infection. Figure 2C shows that, over time, the rate of infected cells does not change, both for the Rad18^+/+^ cells and for the Rad18^−/−^ cells, indicating that Rad18 is dispensable for stable retroviral integration and that infection is not cause of widespread cell death, as confirmed by microscopy inspection (unpublished data).

### Rad18 Affects the Accumulation of Retroviral DNA

**Figure 3:**
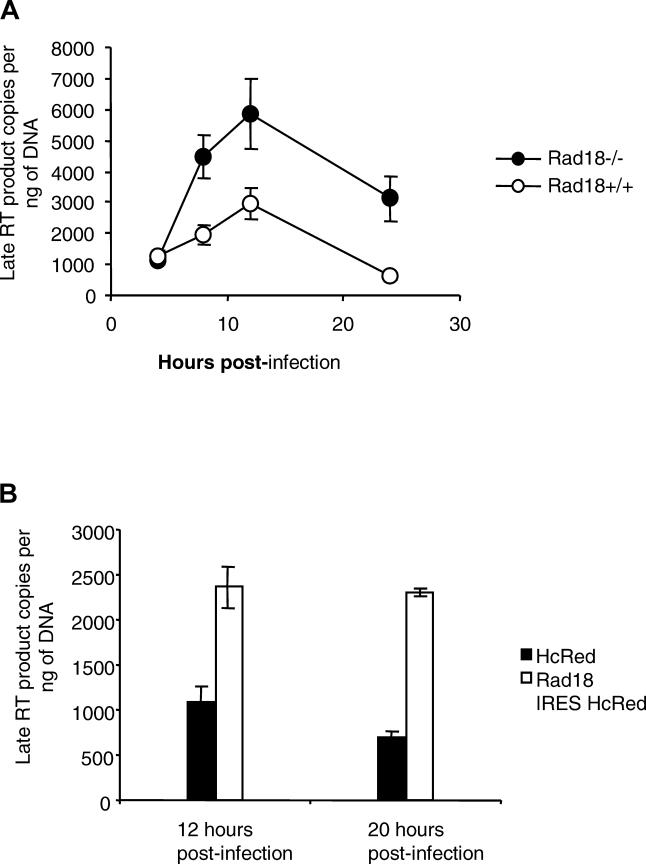
Rad18 Affects the Accumulation of Retroviral DNA (A) MEFs Rad18^−/−^ infected with HIV-1–based EGFP reporter virus present a higher amount of reverse transcription product as compared to wild-type cells. Cells were infected with 12,500 IU of a VSV-G pseudotyped HIV-1 integration-deficient reporter virus for 4 h, and late reverse transcripts were assessed by QPCR at 4, 8, 12, and 24 h after infection. (B) Hela cells transfected with Rad18-IRES-HcRed and IRES-HcRed control plasmids were infected with 12,500 IU of a VSV-G pseudotyped HIV-1 integration-deficient reporter virus. HcRed-positive cells were then sorted 12 and 20 h post-infection, and late reverse transcripts were assessed by QPCR.

To test the presence of an increased accumulation of viral cDNA in Rad18^−/−^ cells, we measured the levels of late reverse transcription products. Cells transduced with VSV-G pseudotyped integration-deficient HIV-1 retrovirus were harvested at different time points and late reverse transcription was estimated by quantitative PCR (QPCR). Figure 3A shows that cDNA peaks between 8 and 12 h post-infection and that Rad18-negative fibroblasts accumulate 2- to 5-fold more viral cDNA at 12 and 24 h than Rad18^+/+^ cells (*t* test *p-*value = 0.05 and 0.013, respectively).

In order to measure the levels of viral cDNA synthesis in the presence of overexpressed Rad18, Hela cells transfected with Rad18-IRES HcRed-expressing plasmid or HcRed control were infected with VSV-G pseudotyped integration-deficient HIV-1 retrovirus and sorted at 12 and 20 h post-infection. Figure 3B shows that Rad18-expressing Hela cells accumulate 2.2- to 3-fold more cDNA as compared to control cells (*t* test *p-*value = 0.02 and 0.014, respectively). This result is seemingly at odds with that observed with the Rad18^−/−^ MEFs in which the absence of Rad18 increased the accumulation of viral cDNA. However overexpression of Rad18 likely results in the titration of other components of Rad18-containing complexes, including those that influence retroviral cDNA metabolism. The saturation of these degradation complexes may preclude their contact with the incoming viral DNA and thereby result in the observed increased reverse transcription product accumulation. Nonetheless, both under- or overexpression of Rad18 affect both reverse transcription and infection, and those findings suggest that Rad18 modulates either the reverse transcription reaction or the stability of the reverse transcription product.

### Increased Adenovirus Infection in Rad18-Negative Cells

**Figure 4:**
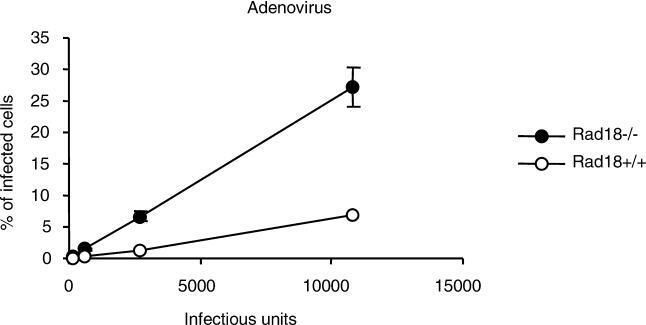
Increased Adenovirus Infection in Rad18-Negative Cells Rad18^−/−^ MEFs show a greater susceptibility to infection with adenovirus expressing EGFP as a reporter as compared to wild type. Cells were infected with serial dilutions of adenovirus EGFP and assessed by flow cytometry 40–44 h after infection. Virus titers (IU) were measured in MEFs Rad18^−/−^.

Because the effect of Rad18 on retroviral infection seemed to involve viral cDNA accumulation, we next asked whether this was due to an effect on reverse transcription or, alternatively, on the degradation of its product. To address this question, we infected both RAD18^+/+^ and Rad18^−/−^ cells with a recombinant replication-defective adenovirus expressing EGFP as reporter. As can be seen in Figure 4, Rad18^−/−^ cells are 4- to 5-fold more sensitive to adenovirus infection than Rad18^+/+^ controls (*t* test *p*-value = 0.02 and 0.019, respectively). Since adenoviruses replicate independently of reverse transcription and integration, this finding suggests that the Rad18 effect on both adeno- and retro-virus infections is the consequence of a response to the presence of foreign DNA rather than the inhibition of reverse transcription.

## Discussion

Prompted by the interaction of the HIV-1 integrase with DNA repair molecule Rad18 [10], we set out to study the effect of Rad18 on retroviral infection.

Surprisingly, cells that lack Rad18 were consistently more susceptible to viral infection than their Rad18^+/+^ controls. Moreover, Rad18-negative cells were not only more sensitive to HIV-1, but also to MLV and adenovirus. Notably, the increase of infection efficiency of the Rad18^−/−^ cells is integration independent, as the effect also occurs using HIV-1 lacking functional integrase. The absence of strong specificity suggests that the apparent anti-viral activity of Rad18 targets a common component in the early phases of infection of all three types of viruses, probably double-stranded DNA. This notion is consistent with the finding that the abundance of viral cDNA product was higher in the Rad18^−/−^ cells. The observation that differences in the accumulation of cDNA become evident after the eighth hour post-infection suggests that Rad18 inhibitory effect may be detectable only when the reverse transcription product has crossed the nuclear envelope and can therefore come in contact with this nuclear protein. Furthermore, this could also explain some of the earlier phenotypes observed in the original RAD18^−/−^ embryonic stem cells [19]. In these cells, a substantial increase in the number of stable transfectants was observed, an outcome that could reflect an altered fate of the incoming DNA

The fact that a DNA repair protein such as Rad18 suppresses retroviral infection instead of aiding its establishment is only partially counterintuitive. Indeed, studies in yeast have reported that a number of molecules involved in DNA repair and genome stability have an inhibitory effect on retrotransposition [22,23]. Furthermore, studies in mammalian cells suggest a protective role for DNA repair molecules against viruses. Rad52, a constituent of the HR system important in the repair of double-strand breaks, has recently been reported to suppress HIV-1 infection [9], as have nucleotide excision repair helicases XPB and XPD [24]. Additionally, the antiviral activity of APOBEC3G [25] is thought to involve a rapid degradation of reverse transcribed viral cDNA [26], and this is proposed to be the result of the sequential activity of enzymes such as UNG2 and APE1, both components of the base excision repair DNA repair pathway. Finally, formation of retroviral DNA circles, promoted by components of the non-homologous end joining for the two–long terminal repeat (2LTR) circles [8], and the HR Mre11 for the one–long terminal repeat (1LTR) circles [4], could be considered the result of an anti-viral activity by these two DNA repair pathways, rather than merely infection byproducts.

In principle, one might therefore consider DNA repair molecules as components of a broader intrinsic immunity to viruses [27]. Within the preintegration phase of retroviral infection, for example, several steps are potential targets for intracellular immunity. The APOBEC3G system targets the nascent retroviral cDNA; Rad52, XPB, XPD, and Rad18 may target the linear cDNA, potentially mediating its degradation; and finally, non-homologous end joining and Mre11 induce the circularization of the remaining viral genome, preventing it from being a substrate for integration. Many of the mechanisms involved are still unclear, but the emerging picture is that the maintenance of genome integrity by DNA repair systems has significant consequences for viral parasitism.

## Materials and Methods

### Cells and cell lines.

Rad18^−/−^ and Rad18^+/+^ MEFs established from Rad18^−/−^ knockout and Rad18^+/+^ mice were described earlier [16]. MEFs, Hela, and HEK 293T cells were cultured in DMEM 10% FCS.

### Viral vectors, expression plasmids, and virus production.

Retroviruses were produced by three plasmid transfection of either HIV-1 or MLV EGFP-bearing vectors in conjunction with Gag-Pol–expressing plasmids and a third plasmid encoding the appropriate envelope protein. HIV-1 reporter vector pHR SIN CSGW was described earlier [20]. HIV-1 Gag-Pol polyprotein was expressed from expression vector pNL/R7 g-p. MLV reporter vector was constructed by cloning EGFP cDNA into the pLNCX2 (Clontech, Palo Alto, California, United States) vector. VSV-G was expressed from plasmid phCMV G [28]. MLV Gag-Pol polyprotein, expressed from plasmid phCMV-intron Gag-Pol, is a gift from François-Loic Cosset (LVRTG, ENS de Lyon–U412 INSERM, Lyon, France). Plasmid FB MO SALF encoding the MLV ecotropic envelope was described before [29].

HEK 293T cells were transfected using Lipofectamine 2000 (Invitrogen) or polyethylenimine (PEI) (Polysciences, Warrington, Pennsylvania, United States) [30]. About 40 h after transfection, supernatants were harvested; 0.45 μm was filtered, aliquoted, and frozen at −70 °C. Retroviruses were titrated for infectivity in NIH3T3 CycT [31]. HIV-1 was also quantitated by p24 enzyme-linked immunosorbent assay (ELISA) performed using the HIV-1 p24 Antigen EIA kit (Beckman Coulter, Allendale, New Jersey, United States).

Adenovirus reporter vector pAdenoVatorΔE1/E3 CMV-5 EGFP, containing the genome of adenovirus serotype 5 (Ad5) with deleted E1 and E3, was constructed by cloning EGFP cDNA into pAdenoVator-CMV-5 (Qbiogene, Irvine, California, United States) and then inserting the adenovirus CMV-5 EGFP containing region of the construct into pAdenoVatorΔE1/E3 by homologous recombination through transformation of Escherichia coli strain BJ5153 as recommended by the manufacturer.

EGFP reporter adenovirus was produced by transfection of pAdenoVatorΔE1/E3 CMV-5 EGFP and serial amplification in HEK 293A (Qbiogene) following the manufacturer's recommendations.

The bicistronic Rad18 vector consists of the human version of an N-terminal FLAG-tagged Rad18 followed by an EMCV IRES and HcRed sequence as reporter.

### Infections and flow cytometry analysis.

Rad18^−/−^ and Rad18^+/+^ MEFs were plated in 24-well plates at 40,000 cells/well, and infected with serial dilutions of the various viruses. Infections were carried out overnight and, in the case of the retroviruses, in the presence of 8 μg/ml of polybrene. About 44 h after infection, cells were trypsinized, fixed with PBS 3% formaldehyde (Tousimis, Rockville, Maryland, United States), and analyzed for EGFP expression by flow cytometry using FACScalibour instrument (Becton Dickinson, Palo Alto, California, United States); 20,000 events were acquired per sample.

For transfection/infection experiments, Hela cells were transfected with plasmids encoding Rad18-IRES-HcRed or IRES-HcRed only, as control, using Lipofectamine 2000 (Invitrogen, Carlsbad, California, United States). Twenty-four hours after transfection, cells were infected with serial dilutions of HIV-1–derived EGFP reporter virus. After 48 h, cells were analyzed by flow cytometry and infection was determined as the percentage of HcRed-positive cells expressing the viral-derived EGFP reporter molecule.

### Quantitative PCR.

Rad18^−/−^ and Rad18^+/+^ MEFs were plated in 24-well plates at a density of 40,000 cells/well. The following day, cells were infected with VSV-G pseudotyped HIV-1 integration-deficient reporter virus previously treated with 150 units/ml of DNase I (Roche, Indianapolis, Indiana, United States) for 30 min at 37 °C. Infections were carried out for 4 h in the presence of 8 μg/ml of polybrene, then cells were rinsed with PBS and fed with complete medium. Cells were harvested at time points 4-, 8-, 12-, and 24-h post-infection, pelleted, and frozen at −70 °C. In the case of the transfection/infection experiments, Rad18-IRES-HcRed and IRES-HcRed control transfected cells were infected with DNase I–treated VSV-G pseudotyped HIV-1 integration-deficient reporter virus. Twelve and twenty hours post-infection, HcRed-positive cells were sorted with MoFlo cell sorter (Cytomation, Fort Collins, Colorado, United States), pelleted, and frozen at −70 °C. Total genomic DNA was extracted using QIAamp DNA Blood kit (Qiagen, Valencia, California, United States) and eluted in 100-μl final volume; 2 μl of each sample were used in order to perform QPCR. Primers used for the detection of late reverse transcription products were the following: primer 1002 91–119 TCTCTGGCTAACTAGGGAAC and 950 339–320 GCCGCCCCTCGCCTCTTG. Reporter fluorophore for the labeling of double-stranded DNA product was SYBR green (Molecular Probes, Eugene, Oregon, United States) and reactions and acquisitions were performed using ABI PRISM 7700 Sequence Detector (Applied Biosystems, Foster City, California, United States) and 7500 Real Time PCR System (Applied Biosystems). Normalization was accomplished by measuring the DNA concentration of each sample using PicoGreen dsDNA Quantitation Reagent (Molecular Probes) following manufacturers instructions. Data are presented as number of late reverse transcription molecules per nanogram of total genomic DNA.

## Supporting Information

### Accession Numbers

The GenBank (http://www.ncbi.nlm.nih.gov/Genbank) accession numbers for the genes and gene products discussed in this paper are Homo sapiens apolipoprotein B mRNA editing enzyme, catalytic polypeptide-like 3G (APOBEC3G) (NM_021822), proliferating cell nuclear antigen (PCNA) (NM_182649), polymerase (DNA directed), eta (POLH) (polymerase η) (NM_006502), RAD18 homolog (NM_020165), RAD52 homolog variant alpha (NM_002879), mRNA for uracil-DNA glycosylase (UNG2) (HS09008), excision repair cross-complementing rodent repair deficiency, complementation group 3 (XPB) (NM_000122), excision repair cross-complementing rodent repair deficiency, complementation group 2 (XPD) (NM_000400), and Mus musculus RAD18 homolog (NM_021385).

## Abbreviations

EGFP: enhanced green fluorescent protein
HEK: human embryonic kidney
HR: homologous recombination
IN: integrase
IU: infectious units
MEF: murine embryonic fibroblast
MLV: murine leukemia virus
QPCR: quantitative PCR
VSV-G: vesicular stomatitis virus protein glycoprotein
